# Fundamental motor skills of kindergarten children in different environments and ethnic groups in Northwest China

**DOI:** 10.1186/s12887-022-03497-7

**Published:** 2022-07-18

**Authors:** Xiaohui Xia, Liang Chao, Chen Nan, Xuejuan Yin, Huifang Zheng, Sheping Zhang

**Affiliations:** 1 Physical Education College, Lanzhou City University, Lanzhou, China; 2 Nantong Haimen Normal Affilicated Primary School, Nantong, China; 3 Sports Department, Lanzhou University of Technology, Lanzhou, China

**Keywords:** Fundamental movement skills, Kindergarten, TGMD-3, Environmental, Ethnic

## Abstract

**Background:**

The status of children’s early motor skills play an important role during childhood and across lifetime. This study described FMS proficiency among boys (*n* = 189) and girls (*n* = 179) kindergarten children from 3 to 6 years old (4.4 s 0.7, mean ± SD) in northwest China. The differences in FMS proficiency of boys and girls from different environments, ethnic groups were analyzed respectively.

**Methods:**

TGMD-3 was used to assess FMS. FMS mastery level was defined according to the correct performance of all criteria over two trials. The correlation between BMI and FMS and the interaction of environmental and ethnic on FMS were analyzed. The general linear model was used to evaluate the differences of boys and girls among environment groups (urban/suburban/county), and ethnic groups (Han/Hui/Tibetan) on the FMS subsets respectively.

**Results:**

FMS proficiency was assessed in 368 3- to 6-year-old children (*n* = 156 urban, *n* = 101 suburban, *n* = 111 county)/(*n* = 208 Han, *n* = 107 Hui, *n* = 53 Tibetan). Overall, the highest skill performance was the run, with 86% achieving mastery level, and the poorest performance was the FH strike, at only 19%. Correlation between BMI and FMS is minimal. According to TGMD-3 scores, there was no significant difference between boys and girls in total FMS (*p* = 0.38). In terms of locomotor skills, boys performed better than girls in the hop, skip and slide (*p* < 0.05). County children performed significantly difference than urban and suburban children. Some skills performed less proficiently, (boys in 6 of 13 skills: run, HJ, slide, TH strike, FH strike and kick; girls in 4 of 13 skills: run, slide, TH strike and kick) and some skills performed more proficiently (boys in dribble; girls in hop and dribble). Tibetan children performed significantly difference than Han and Hui children. Some skills performed less proficiently, (boys in 6 of 13 skills: run, HJ, slide, TH strike, FH strike and kick; girls in TH strike) and some skills performed more proficiently (boys and girls were all in dribble).

**Conclusion:**

Children in northwest China showed certain characteristics in FMS, the county/Tibetan boys and girls performed poorer than others in ability to execute particular process characteristics of some skills and performed more outstanding in other skills. It suggests that a certain group population may need specific focus on interventions to improve their FMS level.

## Background

As basic observable exercise patterns, fundamental movement skills (FMS) lay the foundation for developing of more advanced skills required for recreational and competitive form of physical activities [1]. Status of children’s early motor skills plays an important role during childhood and across lifetime and affects greatly in shaping patterns of movement. Children (2–7 years old) acquire some fundamental skills from reflexive and rudimentary exercise, and obtain more advanced skills within sport-specific-stage [2]. A fully-developed FMS, especially object control skills, are essential for learning more complex sports patterns and increasing the possibility of successfully participating in variable sports in the future. To the contrary, if the defects of FMS are not identified at an early age, children may experience lifelong motor skill problems [3].

Although children have the potential to master most FMS, their mastery is highly individual. It should be noted that these basic sports patterns are not naturally acquired in the process of maturity, and many motor skills are highly influenced by the contextual and family factors that lead to a change in lifestyle, such as unsupervised free time, different forms of outdoor play, and so on [4]. For example, children from families with high socio-economic status are obviously superior to children with middle and/or low socio-economic status in total, fine and gross motor proficiency. FMS proficiency of preschool children is to a certain extent correlated with healthy weight, higher level of physical activity and improved cognitive outcomes [5, 6]. Another aspect, a research on the influence of residence on children’s FMS proficiency showed that there was no difference between urban and rural preschool children [7].

Few studies investigated the FMS proficiency of preschool children in low- and middle-income countries. Children from deprived backgrounds may influenced by the delayed development of motor skills, and thus poorer health. The research results of ethnic differences in sports skills showed that those ethnic groups with physical health risks may have poorer sports skills, and complex association between ethnicity and socio-economic status may further strengthen these risks of health and delays of sports ability [8].

Whether children from China with different living environments or ethnic backgrounds have varying motor skills has not been determined. Population from different regions and ethnics have many differences in economic level, transportation, culture, lifestyle, etc. We assume that there are differences in FMS among people of different environments and ethnics in China. This study aimed to identify FMS proficiency levels of kindergarten children from northwest China (3 to 6 years old) as well as differences in FMS proficiency of boys and girls from variable environments, and ethnic groups.

## Methods

The local education department selected three kindergartens by handy sampling method to participate in the survey. A sample of 368 preschool children (3 to 6 years old) was recruited. According to the living environment, children were divided into urban, suburban, and county groups. 156 urban children group (74 girls and 82 boys), children living in Lanzhou, capital of Gansu province, northwest China. The city is modern, and its citizens live in a fast pace. The conomic level of lanzhou is higher than other regions. Han ethnic is the majority, followed by Hui. The second group was composed of 111 suburban children (53 girls and 58 boys). They living in the country side, and most of their parents are farmers, who are engaed in agricultural cultivation. Almost all the suburban children are Han ethnic. The third group was composed of 101 children (52 girls and 49 boys). They live in the county, an autonomous region for ethnic minorities in Gansu province. Their parents are farmers, herdsmen or workers, and their life is less stressful and more leisurely. Han and Tibetan are the main ethnic groups in the county.

Data were collected from September 2018 to October 2019. Age, weight, and height details for each child were provided by the children’s kindergarten. FMS proficiency was measured with the Test of Gross Motor Development-edition 3 (TGMD-3), validated in the Chinese polulation [9]. Ethical approval for this research was provided by the Science and Technology Office of Lanzhou City University. All the children in the study participated with signed informed parental consent.

The TGMD-3 involved the assessment of six LM skills: run, gallop, hop, skip, horizontal jump (HJ), and slide; and seven OC skills: two-hands strike (TH strike), forehand strike (FH strike), dribble, two-hands catch (TH catch), kick, overhand throw (OH throw), and underhand throw (UH throw). Each skill was graded according to a set of performance criteria that represent specific components of the skill. There were between 3–5 performance criteria for each skill [10–12]. The researchers were trained and obtained a test qualification permit from the College of Physical Education and Health of Huadong Normal University, a TGMD-3 testing authority. Each test group consisted of 2 children, 2 testers, and 1 photographer. The subject repeated a test twice after observing the tester’s action demonstration.

The duplicate scores of different skills were added to obtain the LC skill score (6 skills, 23 points, with a maximum possible score of 46) and the OC skill score (7 skills, 27 points, with a maximum possible score of 54), the sum of which is the FMS total score (with a maximum possible score of 100).For example, when performing the run skill, the following four points of performance criteria applied: (1) Arms moved in opposition to legs with elbows bent, (2) Brief period where both feet were off the ground, (3) Narrow foot placement landing on heel or toe (not flat-footed), (4) Non-support leg bent about 90 degrees, so the foot is close to the buttocks. Performance criteria for each skill performed correctly were scored as 1, and performance criteria performed incorrectly were scored as 0. The maximum score of the run skill was 8 (repeated twice with a maximum of 4 points each time).

Mastery was defined as the correct performance of all criteria over two trials (e.g., a total score of 8 for the run or a score of 2 for one aspect of performance). Near mastery was defined as the correct performance of all but one performance criteria over two trials or a score of 1 for one aspect of performance. Poor mastery was defined as the incorrect performance/absence of more than one performance criteria over two trials or a score of 0 for one aspect of performance [13, 14].

FMS data was analyzed by using SPSS version 24.0 for Windows. Descriptive statistics for mastery levels, LM skills, OC skills, and FMS were calculated. Data presented as mean ± SD. Bivariate correlation analysis was used to evaluate the relationship between BMI and raw skill scores. The pearson coefficient of correlation (r) determined correlations. The separation of boys from girls, the interaction effect of environmental and ethnic groups on FMS were analyzed by the general linear model. For variables with interaction effect, simple effect analysis was used to compare groups difference. Otherwise, main effect analysis was used. Statistical significance was set at *p* < 0.05. A Bonferroni adjustment of the alpha was calculated at 0.017 (p/3) to control for Type I error.

## Results

### Participant information and mastery levels of FMS

Mean age of participants (boys, *n* = 189; girls, *n* = 179) was 4.3 ± 0.9 years. Children came from three different living environment (urban, *n* = 156; suburban, *n* = 111; county, *n* = 101) and three ethnic (Han, *n* = 208; Hui, *n* = 107; Tibetan, *n* = 53). Participants’ information are showed in Table 1.

**Table 1:** Participant information divided by ethnicity, environment, and gender (mean ± SD)

Ethnicity	Environment	Gender	n	Age (yr.)	Height (cm)	Weight (kg)	BMI
Han *n* = 208	Suburban	Male	56	4.3 ± 0.9	107.4 ± 7	19.3 ± 3.3	16.7 ± 1.5
		Female	53	4.4 ± 0.9	105.7 ± 6.8	17.9 ± 2.7	15.9 ± 1.3
	County	Male	18	4.6 ± 1	110.2 ± 13.4	16.5 ± 1.6	14.1 ± 3.8
		Female	23	4.5 ± 1	108.1 ± 13.5	16.5 ± 1.8	14.7 ± 3.8
	Urban	Male	35	4.4 ± 0.7	111.9 ± 5.4	20.2 ± 2.7	16.1 ± 1.6
		Female	23	4.6 ± 0.6	112.1 ± 6.5	20.1 ± 3.3	16 ± 1.9
Hui *n* = 107	Suburban	Male	2	5 ± 0	109.5 ± 2.1	21.3 ± 1.8	17.7 ± 0.8
	County	Male	5	5 ± 0	124.8 ± 3.3	17.2 ± 2.2	11.1 ± 1.3
		Female	3	5 ± 0	124 ± 3.6	16.3 ± 0.6	10.6 ± 1
	Urban	Male	46	4.6 ± 0.8	114.3 ± 6.2	21.4 ± 3.8	16.3 ± 2
		Female	51	4.3 ± 0.8	109.7 ± 5.9	18.9 ± 2.3	15.7 ± 1.1
Tibetan *n* = 53	County	Male	26	4.3 ± 0.9	106.9 ± 16.3	14.7 ± 3.5	13 ± 1.7
		Female	26	4.5 ± 0.9	113.5 ± 9.9	17.8 ± 4.3	14.4 ± 6.7
	Urban	Male	1	4 ± 0	113.5 ± 0	18.5 ± 0	14.4 ± 0
Total			368	4.4 ± 0.7	110 ± 9.3	18.7 ± 3.5	15.5 ± 2.8

**Fig. 1:**
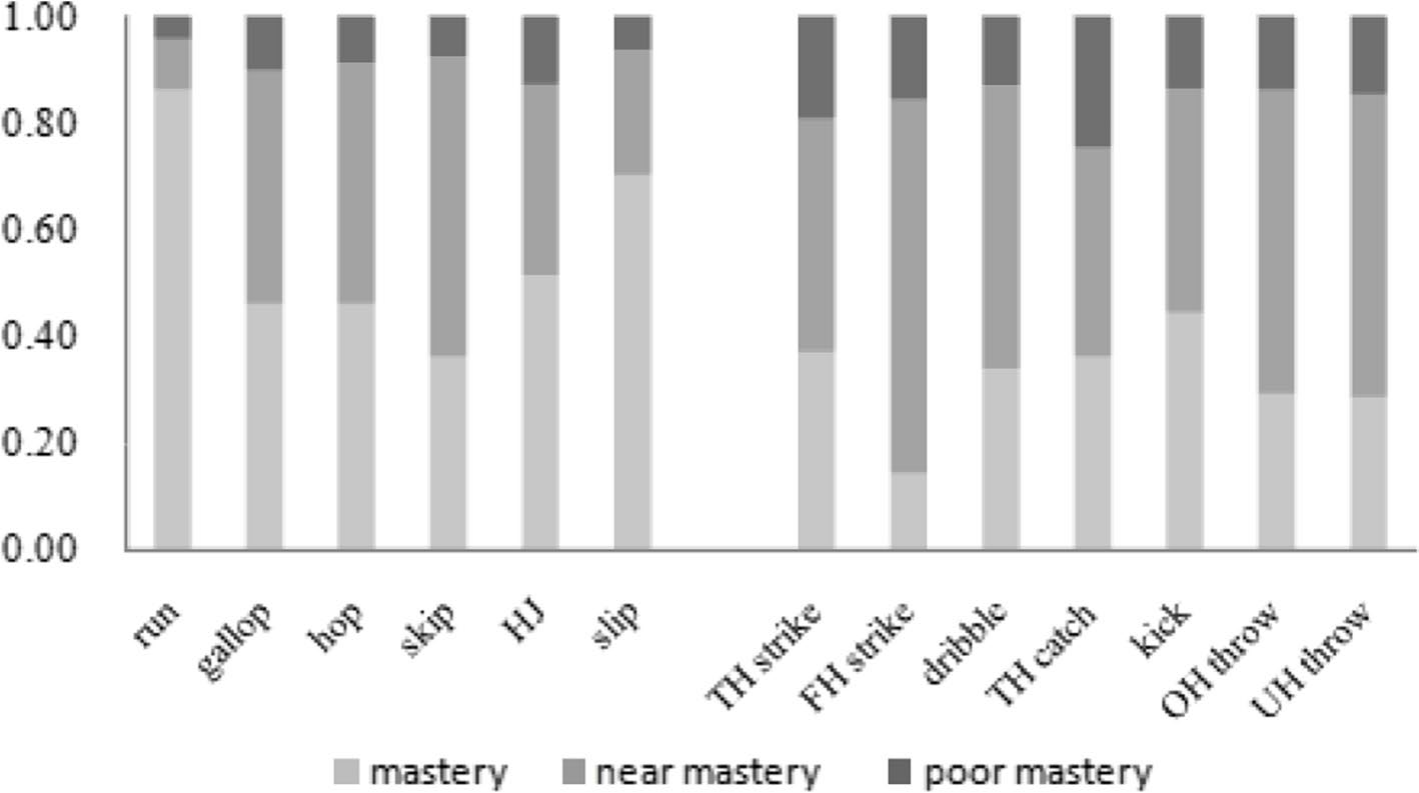
Percentage of participants (*n* = 368) achieving mastery, near mastery, and poor mastery in each skill. HJ = horizontal jump, TH strike = two-hand strike, FH strike = forehand strike, TH catch = two-hand catch, OH = overhand throw, UH throw = underhand throw

In each of these items, the top percent of mastery was for the run (86%) and slide (70%), while the top percent of poor mastery was TH catch (25%) and TH strike (19%). Further analysis of the specific problems found that the highest percent of mastery in the four run parameters was the second point (95%), “brief period where both feet are off the ground.” The higher percent of mastery in the four aspects of slide were given by “slide to right continuously for 4 times” and “slide to the left continuously for 4 times”, which were 78% and 79%, respectively. The percent of poor mastery level of the 3 points of TH catch skill was consistently low: child’s hands are positioned in front of the body with the elbows flexed (25%), arms extend reaching for the ball as it arrives (26%), and the ball is caught by hands only (22%). In the five points of TH strike, the highest percentage of poor mastery was given to “this ball sending it straight ahead,” as 33% of children missed the ball completely (Fig. 1).

### BMI and gender influence on FMS proficiency

Results of Pearson analyses on the correlation between BMI and 13 FMS skills showed that only run (*p* < 0.05, *r* = 0.19) and Skip (*p* < 0.05, *r* = 0.14) skills had significant correlation with BMI, indicating that the correlation between BMI and FMS is minimal.

Results of the t-test analyses indicated the impact of gender (girls/boys) on individual skill. Scores are summarized in Table 2. No significant difference in FMS or OC was detected between boys and girls. There was a significant difference in LM between girls and boys (*p* = 0.01, *F* = 7.04). Girls were better than boys in the hop (*p* = 0.01, *F* = 6.89), skip (*p* < 0.001, *F* = 16.53) and slide (*p* = 0.02, *F* = 5.91). Because of the significant differences between boys and girls in certain FMS skills, in order to exclude the influence of gender, boys and girls were studied separately when studing the influene of environmental and ethnic differences on FMS.

**Table 2:** Between-gender differences for individual skills

	Total (*n* = 368)	Boys (*n* = 189)	Girls (*n* = 179)	p	F
Run (range 0–8)	7.1 ± 1.6	6.9 ± 1.8	7.2 ± 1.4	0.11	2.47
Gallop (range 0–8)	4.1 ± 2.9	4 ± 2.8	4.2 ± 3	0.67	0.19
Hop (range 0–8)	4 ± 2.9	3.7 ± 2.8	4.4 ± 2.9	0.01	6.89
Skip (range 0–6)	2.4 ± 2.4	1.9 ± 2.3	2.9 ± 2.5	< 0.001	16.53
HJ (range 0–8)	4.6 ± 2.2	4.7 ± 2.2	4.5 ± 2.2	0.41	0.68
Slide (range 0–8)	5.9 ± 2.5	5.6 ± 2.7	6.2 ± 2.3	0.02	5.91
LM (range 0–46)	28.1 ± 9.6	26.9 ± 9.5	29.5 ± 9.6	0.01	7.04
TH strike (range 0–10)	4.7 ± 2.8	4.6 ± 2.9	4.8 ± 2.7	0.42	0.66
FH strike (range 0–8)	1.8 ± 2	1.9 ± 2	1.7 ± 1.9	0.20	1.65
Dribble (range 0–6)	2.4 ± 2.2	2.5 ± 2.2	2.4 ± 2.2	0.82	0.05
TH catch (range 0–6)	2.9 ± 1.4	3 ± 1.4	2.8 ± 1.5	0.34	0.90
Kick (range 0–8)	4.1 ± 2.4	4.2 ± 2.5	4 ± 2.3	0.45	0.56
OH throw (range 0–8)	2.9 ± 2.4	3 ± 2.4	2.8 ± 2.4	0.61	0.26
UH throw (range 0–8)	2.9 ± 2.9	3.1 ± 2.8	2.7 ± 2.7	0.16	1.95
OC (range 0–54)	21.7 ± 9.6	22.1 ± 9.6	21.2 ± 9.7	0.35	0.87
FMS (range 0–100)	49.8 ± 17.4	49 ± 17.4	50.7 ± 17.3	0.35	0.88

Data presented as mean ± SD; *p*-values showed for differences between genders using the t-test

*HJ* Horizontal jump, *TH strike* Two-hand strike, *FH strike* Forehand strike, *TH catch* Two-hand catch, *OH* Overhand throw, *UH throw* Underhand throw, *LM* Locomotor, *OC* Object control, *FMS* Foundamental movement skill

### Environmental and ethnic differences in FMS proficiency

The interaction effect between environment and ethnic was analyzed. Results showed that there was interaction effect on gallop (*p* = 0.00, η^2^ = 0.07) of boys, gallop (*p* = 0.01, η^2^ = 0.04) and FH Strike (*p* = 0.04, η^2^ = 0.03) of girls. There was no interaction between environment and ethnic in other skills. When analyzing the relationship between environment/ethnic and FMS, the interactive skills between environment and ethnic groups (gallop of boys, gallop and FH strike skills of girls) were analysed by the simple effect analysis, and others were analysed by the main effect analysis.

#### Environmnetal differences in FMS proficiency

In comparing the 3 different environmental groups, there were significant differences among urban, suburban, and county boy groups in FMS, OC, and LM scores. Analyses indicated that county boys had a significantly less sum of FMS, OC, and LM scores than suburban and county children (all *p* = 0.00). The Bonferroni method was used to adjust the significance level of the paired test. The differences in FMS, OC, and LM scores between urban vs. county boy groups and suburban vs. county boy groups (all adjusted *p* = 0.00) (Table 3).

**Table 3:** Enviornmental differences for individual skills

	boys								girls							
	Urban (*n* = 82)	County (*n* = 49)	Suburban (*n* = 58)	p	F	p^a^	p^b^	p^c^	Urban (*n* = 74)	County (*n* = 52)	Suburban (*n* = 53)	p	F	p^a^	p^b^	p^c^
Run (range 0–8)	7.62 ± 0.75	5 ± 2.4	7.64 ± 0.74	0.00	66.13	0.00	0.99	0.00	7.66 ± 0.73	6.48 ± 1.99	7.3 ± 1.15	0.00	12.24	0.00	0.40	0.01
Gallop (range 0–8)	4.52 ± 2.98	3.02 ± 3	4.22 ± 2.27	0.10	2.32				4.14 ± 3.27	4 ± 3.17	4.4 ± 2.28	0.12	2.18			
Hop (range 0–8)	3.96 ± 3.02	3.59 ± 2.76	3.31 ± 2.64	0.40	0.92				4.53 ± 3.18	5.35 ± 2.66	3.45 ± 2.23	0.00	6.13	0.32	0.10	0.00
Skip (range 0–6)	2.17 ± 2.53	1.43 ± 1.99	1.95 ± 2.14	0.20	1.63				3.12 ± 2.58	3.04 ± 2.58	2.51 ± 2.14	0.35	1.05			
HJ (range 0–8)	5.39 ± 2.07	3.73 ± 2.08	4.6 ± 2.25	0.00	9.40	0.00	0.10	0.11	4.78 ± 2.25	4.1 ± 2.21	4.6 ± 2.05	0.21	1.56			
Slide (range 0–8)	6.29 ± 2.64	4.47 ± 2.26	5.47 ± 2.81	0.00	7.60	0.00	0.20	0.15	6.76 ± 2.29	5.31 ± 2.49	6.32 ± 2.03	0.00	6.28	0.00	0.87	0.07
LM (range 0–46)	29.96 ± 9.16	21.24 ± 9.17	27.19 ± 8.01	0.00	15.03	0.00	0.21	0.00	30.99 ± 9.93	28.27 ± 10.44	28.58 ± 7.97	0.23	1.71			
TH strike (range 0–10)	5.41 ± 2.59	2.59 ± 2.66	4.98 ± 2.61	0.00	19.00	0.00	1.00	0.00	5.57 ± 2.36	3.27 ± 3.08	5.19 ± 2.23	0.00	13.27	0.00	1.00	0.00
FH strike (range 0–8)	2.06 ± 2.21	1.69 ± 1.52	1.9 ± 2.16	0.01	4.83	0.00	0.04	0.66	1.82 ± 2	1.62 ± 1.59	1.45 ± 1.96	0.54	0.62			
Dribble (range 0–6)	1.88 ± 2.03	4.27 ± 1.54	1.74 ± 2.19	0.00	28.10	0.00	1.00	0.00	1.81 ± 2.17	3.96 ± 1.64	1.7 ± 2.15	0.00	21.75	0.00	1.00	0.00
TH catch (range 0–6)	3.22 ± 1.27	2.76 ± 1.42	2.83 ± 1.51	0.11	2.22				2.96 ± 1.43	2.92 ± 1.52	2.58 ± 1.42	0.32	1.15			
Kick (range 0–8)	5.27 ± 2.13	1.82 ± 2.04	4.69 ± 1.78	0.00	48.12	0.00	0.28	0.00	4.64 ± 2.4	3.04 ± 2.31	4.09 ± 1.62	0.00	8.33	0.00	0.50	0.04
OH throw (range 0–8)	3.41 ± 2.33	2.43 ± 2.42	2.78 ± 2.49	0.06	2.84				3.11 ± 2.19	3.04 ± 2.7	2.25 ± 2.43	0.11	2.23			
UH throw (range 0–8)	2.77 ± 2.75	2.67 ± 2.74	3.86 ± 2.9	0.04	3.29	1.00	0.07	0.09	2.77 ± 2.58	2.77 ± 3.15	2.45 ± 2.44	0.78	0.25			
OC (range 0–54)	24.02 ± 9.34	18.22 ± 8.09	22.78 ± 10.24	0.00	6.13	0.00	1	0.04	22.68 ± 10.38	20.62 ± 8.87	19.72 ± 9.25	0.21	1.59			
FMS (range 0–100)	53.99 ± 16.66	39.47 ± 15.71	49.97 ± 16.73	0.00	12.11	0.00	0.47	0.00	53.66 ± 18.42	48.88 ± 17.84	48.3 ± 14.78	0.15	1.89			

Data are presented as mean ± SD; p^a^: Urban vs. County; p^b^: Urban vs. Suburban; p^c^: County vs. Suburban. p^a^, p^b^, p^c^ showed for Bonferroin adjusted Sig

*HJ* Horizontal jump, *TH strike* Two-hand strike, *FH strike* Forehand strike, *TH catch* Two-hand catch, *OH* Overhand throw, *UH throw* Underhand throw, *LM* Locomotor, *OC* Object control, *FMS* Foundamental movement skill

As for boys, when comparing raw scores of specific skill, 3 LM skills (run, HJ, and slide) and 5 OC skills (TH strike, FH strike, dribble, kick and UH throw) all showed a significant difference among urban, county and suburban groups (all *p* < 0.05). After adjusting the significance level using the Bonferroin method, it was found that 6 skills (run, HJ, slide, TH strike, dribble, kick) with statistically significant differences were among urban groups vs. county groups and county groups vs. suburban groups. FH strike with a statistically significant difference was among urban groups vs. county groups and urban groups vs. suburban groups. The county group scored highest in dribble skill and the suburban groups scored highest in run and UH throw skills, while the urban group scored highest in the remainder of the skills. County boys performed significantly less proficiency than urban and suburban boys in FMS, specifically in 6 of the 13 skills: run, HJ, slide, TH strike, FH strike and kick, while they performed more proficiency than urban and suburban boys in dribble.

As for girls, when comparing raw scores of specific skills, 3 LM skills (run, hop, slide) all showed a significant difference among the 3 groups (all *p* < 0.05). After adjusting the significance level using the Bonferroni method, it was found that the skills and groups with statistically significant differences were as follows. Run: urban group vs. county group, county group vs. suburban group; hop: county group vs. suburban group; slide: urban group vs. county group (all adjusted *p* < 0.05). 3 OC skills, that is, TH strike, dribble and kick, all showed a significant difference among the 3 groups (all *p* < 0.05). After adjusting the significance level using the Bonferroni method, it was found that the skills and groups with statistically significant differences was among urban groups vs. county groups and county groups vs. suburban groups (all adjusted *p* < 0.05). The county group scored highest in hop and dribble skills and the suburban group scored highest in gallop skill, while the urban group scored highest in the remainder of the skills. County girls performed significantly less proficiency than urban and suburban boys in FMS, specifically in 4 of the 13 skills: run, slide, TH strike and kick, while they performed more proficiency than urban and suburban girls in FMS, specifically in 2 of the 13 skills: hop and dribble.

#### Ethnic differences in FMS proficiency

In comparing the 3 different ethnic groups, there were significant differences among Han, Hui and Tibetan boy groups in FMS, OC scores. Analyses indicated that Tibetan boys had a significantly less sum of LM and FMS scores than Han and Hui boys (*p* = 0.00, *p* = 0.01, respectivly). After adjusting the significance level using the Bonferroni method, it was found that the scores and groups with statistically significant differences were as follows. LM: Han vs. Tibetan boy groups and Hui vs. Tibetan boy groups (adjusted *p* = 0.02, *p* = 0.00, respectivly), FMS: Hui vs. Tibetan boy groups (adjusted *p* = 0.00) (Table 4).

**Table 4:** Ethnic differences for individual skills

	boys								girls							
	Han (*n* = 109)	Hui (*n* = 53)	Tibetan (*n* = 27)	p	F	p^a^	p^b^	p^c^	Han (*n* = 99)	Hui (*n* = 54)	Tibetan (*n* = 26)	p	F	p^a^	p^b^	p^c^
Run (range 0–8)	7.27 ± 1.43	7.34 ± 1.29	4.89 ± 2.5	0.00	26.45	1.00	0.00	0.00	7.17 ± 1.48	7.48 ± 0.99	6.81 ± 1.77	0.12	2.12			
Gallop (range 0–8)	3.96 ± 2.72	4.85 ± 2.88	2.78 ± 2.83	0.35	1.06				4.14 ± 2.72	4.22 ± 3.31	4.19 ± 3.25	0.13	2.03			
Hop (range 0–8)	3.52 ± 2.78	3.83 ± 2.99	3.93 ± 2.85	0.71	0.34				4.17 ± 2.69	4.44 ± 3.16	5.5 ± 2.69	0.11	2.25			
Skip (range 0–6)	1.94 ± 2.29	1.91 ± 2.34	1.81 ± 2.25	0.97	0.03				2.92 ± 2.28	2.69 ± 2.66	3.38 ± 2.7	0.49	0.71			
HJ (range 0–8)	4.83 ± 2.25	5 ± 2.04	3.7 ± 2.27	0.03	3.48	1.00	0.05	0.04	4.38 ± 2.22	4.63 ± 2.15	4.88 ± 2.16	0.54	0.62			
Slide (range 0–8)	5.44 ± 2.8	6.45 ± 2.27	4.33 ± 2.51	0.00	6.15	0.07	0.15	0.00	6.08 ± 2.47	6.61 ± 2.26	5.85 ± 1.95	0.29	1.26			
LM (range 0–46)	26.96 ± 8.93	29.38 ± 8.99	21.44 ± 10.54	0.00	6.69	0.36	0.02	0.00	28.87 ± 9.28	30.07 ± 10.13	30.62 ± 9.73	0.06	2.91			
TH strike (range 0–10)	4.71 ± 2.82	5.26 ± 2.6	2.52 ± 2.64	0.00	9.43	0.67	0.00	0.00	5.15 ± 2.5	5.13 ± 2.47	2.69 ± 3.17	0.00	9.88	1.00	0.00	0.00
FH strike (range 0–8)	1.95 ± 2.15	1.87 ± 1.87	1.85 ± 1.9	0.02	3.88	0.68	0.00	0.00	1.72 ± 1.96	1.8 ± 1.9	1.12 ± 1.4	0.28	1.29			
Dribble (range 0–6)	2.08 ± 2.18	2.23 ± 2.22	4.41 ± 1.34	0.00	13.77	1.00	0.00	0.00	2.24 ± 2.24	1.93 ± 2.14	4 ± 1.85	0.00	8.71	1.00	0.00	0.00
TH catch (range 0–6)	2.94 ± 1.49	3.17 ± 1.24	2.74 ± 1.29	0.40	0.92				2.79 ± 1.4	2.94 ± 1.42	2.81 ± 1.74	0.81	0.21			
Kick (range 0–8)	4.22 ± 2.36	5.13 ± 2.11	2.26 ± 2.4	0.00	13.97	0.06	0.00	0.00	3.98 ± 2.17	4.3 ± 2.38	3.54 ± 2.34	0.37	1.01			
OH throw (range 0–8)	3.07 ± 2.39	3.02 ± 2.45	2.41 ± 2.52	0.44	0.83				2.52 ± 2.4	3.07 ± 2.14	3.54 ± 2.98	0.11	2.23			
UH throw (range 0–8)	3.09 ± 2.89	3.26 ± 2.74	2.67 ± 2.81	0.67	0.40				2.65 ± 2.64	2.81 ± 2.61	2.5 ± 3.19	0.88	0.13			
OC (range 0–54)	22.07 ± 9.88	23.94 ± 9.22	18.85 ± 8.38	0.08	2.58				21.04 ± 9.67	21.98 ± 9.99	20.19 ± 9.19	0.72	0.33			
FMS (range 0–100)	49.04 ± 17.29	53.32 ± 16.44	40.3 ± 16.94	0.01	5.25	0.40	0.05	0.00	49.91 ± 17.03	52.06 ± 18.08	50.81 ± 17.38	0.77	0.27			

Data are presented as mean ± SD; p^a^: Han vs. Hui; p^b^: Hui vs. Tibetan; p^c^: Han vs. Tibetan. p^a^, p^b^, p^c^ showed for Bonferroin adjusted Sig

*HJ* Horizontal jump, *TH strike* Two-hand strike, *FH strike* Forehand strike, *TH catch* Two-hand catch, *OH* Overhand throw, *UH throw* Underhand throw, *LM* Locomotor, *OC* Object control, *FMS* Foundamental movement skill

As for boys, when comparing raw scores of specific skills, 3 LM skills (run, HJ and slide) showed a significant difference among the 3 groups (all *p* < 0.05). After adjusting the significance level using the Bonferroni method, it was found that the skills and ethnicities with statistically significant differences were as follows. Run: Han ethnic vs. Tibetan ethnic, Hui ethnic vs. Tibetan ethnic; HJ and slide: Hui ethnic vs. Tibetan ethnic (all adjusted *p* < 0.05). 4 OC skills (TH strike, FH strike, dribble and kick) had significant differences among 3 groups (all *p* < 0.05). After adjusting the significance level using the Bonferroni method, it was found that the skills and ethnicities with statistically significant differences were among Han ethnic vs. Tibetan ethnic and Hui ethnic vs. Tibetan ethnic (all adjusted *p* < 0.05). The Han ethnic group scored highest in skip and FH strike skills and the Tibetan ethnic group scored highest in hop and dribble skills, while the Hui ethnic group scored highest in the remainder of the skills. Tibetan boys performed significantly less proficiency than Han and Hui boys in FMS, specifically in 6 of the 13 skills: run, HJ, slide, TH strike, FH strike and kick. while they performed more proficiency than Han and Hui boys in dribble.

As for girls, when comparing raw scores of specific skills, 2 OC skills (TH strike and dribble) had a similar significant differences to boys. The Han ethnic group scored highest in TH strike skills and the Tibetan ethnic group scored highest in hop, skip, HJ, dribble and OH throw skills, while the Hui ethnic group scored highest in the remainder of the skills. Tibetan girls performed significantly less proficiency than Han and Hui girls in TH strike, while they performed more proficiency than Han and Hui girls in dribble.

## Discussion

FMS is a multifactorial performance. Subjects in this study came from different regions and ethnicities. Whether BMI affect motor skills is uncertain. An investigation of 1200 preschoolers between the ages of 3–7 from 12 preschools throughout Taiwan and another report of 216 preschoolers ages of 5–6 Korean children showed that BMI had a minimal influence on FMS skills [15, 16]. In our study, in order to clarify the relationship between BMI and FMS, a correlation analysis was run. Results were consistent with previous researches, and the correlation is very weak.

Some studies have shown that there are gender differences in FMS and that boys score higher than girls [17]. Evidence for gender differences in locomotor skill proficiency is inconsistent, with some studies reporting girls superiority [18–21] and others supporting the current findings of no significant differences between boys and girls [22, 23]. A Japanese study on 60 healthy 5-year-old children showed that girls had significantly better locomotor skills and boys had significantly better object control skills [18]. An English study on children aged 4–7 years old showed that females outperformed males for fine motor skills and boys outperformed girls for catch and dribble gross motor skills [6]. The possible reason is that at the age of 3 to 6, males and females are biologically similar, but females are more likely to disengage from these highly competitive activities due to perceived sex roles and the idea that they should act in a more caring, less competitive manner [14]. Therefore, the performance of specific motor skills is different. In our study, girls were significantly better than boys in locomotor skills: hop, skip and slide. Therefore, when studying the influence of environment/ethnic on FMS, subjects were divided into boys group and girls group.

In this study, the FMS performance of children varied among environments. FMS scores of children in county is less than others. This finding is consistent with research from other countries [24–27]. Study showed that rural low-income children performed significantly better than urban high-income and urban low-income children (*p* = 0.028 and *p* = 0.009, respectively). Another study focused on socioeconomic and familial factors and found that children living below the poverty threshold were more likely to have better gross motor skills, and that girls have better locomotor skills than boys [21].Children from the countryside potentially spend more time outdoors, whereas children from metropolitan areas most frequently engaged in organized sports [28].

It is not clear whether ethnicity affect the development of FMS in young children. Previous results have been mixed. A 3 yerars longitudinal study to 313 kindergarteners showed that Hispanic children showed a greater increase for sedentary behavior and lower MVPA than non-Hispanic children [29]. Research investigating the FMS of catching, balancing, and jumping in 4- to 12-year-old Euro-American (*n* = 103) and Mexican–American children (*n* = 104) found no significant differences between the two groups in these tasks [30]. The authors concluded that they found no ethnic differences because many of the activities were similar in both cultures during the early childhood years. In contrast, in Eyre’s study, for White and South Asian, at baseline, there were significant differences between ethnicities for run, stationary dribble, throw, roll, 7-skills score, and medicine ball throw. A larger proportion of South Asian children were categorised as poor in motor performance of the skill component and/or higher levels of white children showing mastery of the motor component [16].

Our main findings were most county children’s FMS skills were poorer than those of urban and suburban children. Most Tibetan children’s FMS skills were poorer than those of Han and Hui children. But their dribble skill was better than that of others. Such findings were common between boys and girls. The skill differences among ethnics and regions were quite diverse and the specific reasons were not illustrated in this study. One of the possible reasons was that children in different environments play different games: most urban children enjoyed playing soccer, dancing and other fashionable sports; suburban children played kite, run-and-catch in open areas; and county children liked wrestling, racing, which were popular among Tibaten ethnic.

Compared with girls, boys’ skills were more influenced by regions and ethnics. The skills that were different among girls’ groups were also different among boys' groups. In contrast, there were some skills that were different among boys but there was no difference among girls. From physiological perspective, although there is no obvious physiological difference between boys and girls in early childhood, boys tend to easyily get access to activities and receive encouragements, and have more opportunities to participate in competitive games, which leads to skill performance differences between boys and girls.

## Limitations of this study

Caution is needed to interpret the results, and several limitations should be acknowledged. First, multiple factors may affect the study outcomes. These include the individual characteristics of each child, such as child’s independent walking age, time spent sedentary and outdoors, participation in organized sports activities, and access to electronic devices. Family factors include parents’ education level, physical frequency, and sedentary behavior. Environmental factors mainly refer to the use of sports facilities [31, 32]. The developmental delay in motor competence is also associated with decreased health-enhancing physical activity, physical fitness, perceived competence, and increased obesity [33]. Other factors that may be important that have not been considered or where the data is unavailable include exposures outside of the home, community recreational activities, and genetic predisposition. Nevertheless, research is typically focused on limited factors; no comprehensive study has been conducted. Due to the complexity of influencing factors, the reasons for the related differences among groups would be eplored in futher research.

Another limitation is that only one process-oriented tool was used. Thus, certain FMS (such as stability skills) were not examined. Although the TGMD-3 is a validated tool that has been used in numerous other international studies, further research should consider using more than one tool to evaluate FMS comprehensively.

## Conclusion

In this study, children in northwest China showed certain characteristics in FMS, suggesting that they were influenced by the environment and ethnicity. The county/Tibetan boys and girls performed poorer in ability to execute particular process characteristics of some skills than others and performed more outstanding in other skills. It suggests that a certain group population may need specific focus on interventions to improve their FMS level. Further research will provide greater clarity for improving targeted interventions of FMS.

## Data Availability

The datasets used or analysed the current study are available from the corresponding author on reasonable request.

## Abbreviations

FMS: Fundamental movement skills
TGMD-3: Test of Gross Motor Development-Edition 3
LM: Locomotor
OC: Object control
HJ: Horizontal jump
TH strike: Two-hand strike
FH strike: Forehand strike
TH catch: Two-hand catch
OH: Overhand throw
UH throw: Underhand throw
LM: Locomotor
BMI: Body mass index
